# A very musical psychopathology – from intrusive musical imagery, to musical obsessions and hallucinations

**DOI:** 10.1192/j.eurpsy.2023.2122

**Published:** 2023-07-19

**Authors:** A. S. Morais, F. Martins, P. Casimiro, V. Henriques, N. Descalço, R. Diniz Gomes

**Affiliations:** Psychiatry, Hospital Garcia de Orta, Lisbon, Portugal

## Abstract

**Introduction:**

The semiological spectrum that encompasses musical imagery is a very confusing field, as it is often difficult to understand the nature of the underlying psychopathological phenomenon from the patient’s description.

**Objectives:**

The purpose of the authors is to explore reviewing, distinguishing and organizing the concepts such as Intrusive musical imagery, musical obsessions, musical hallucinations, pseudohallucinations and musical palinacousis.

**Methods:**

A brief non-systematized review is presented, using the literature available on PubMed and Google Scholar.

**Results:**

Intrusive musical imagery (earworms, *ohrwurms*, or involuntary musical imagery) occur in more than 85% of general population, without pathology or ear disease. It involves the involuntary repetition of 15-30 seconds of a fragment of music/tune, persisting like a looping soundtrack, not being aversive.

Musical obsessions are a rare form of intrusive imagery, occurring either with other symptoms of Obsessive Compulsive Disorder or isolated (“The stuck song syndrome”). It is recurrent, persistent, intrusive, unintentional, time consuming and causes distress or functional impairment (although not as ego-dystonic and aversive as usually intrusive visual imagery are); preserved insight.

Musical hallucinations occur only in 0,16% in a general hospital; they can be linked to psychiatric diseases, but they are more common in neurological diseases (cerebral lesions, Parkinson’s disease, delirium, drug induced…). They are reported to with less controllability, less lyrical content, and lower familiarity, than other forms of inner music; are perceived to arise from an external source and are interpreted as veridical.

Musical Pseudohallucinations can arise after severe hearing loss, in hallucinogen intoxication and in psychotic or non-psychotic disorders (as dissociative states or in borderline personality disorder). They occur in inner/subjective space, but insight can fluctuate.

Musical palinacousis is associated with electroencephalogram and neuroimaging abnormalities, linked to structural brain pathology. There is perseveration (echoing) of an external auditory stimulus occurs after cessation of the stimulus.

**Conclusions:**

A rash classification can lead to misdiagnosis (for e.g. interpreting obsessive symptoms as hallucinatory phenomena or rendering an organic pathology undiagnosed) and the institution of inappropriate therapy. It is important to carefully explore these musical imagery phenomena when patients present these complaints, taking some time to characterize them.

**Disclosure of Interest:**

None Declared

